# A new method to predict return of spontaneous circulation by peripheral intravenous analysis during cardiopulmonary resuscitation: a rat model pilot study

**DOI:** 10.1186/s40635-024-00679-8

**Published:** 2024-11-12

**Authors:** Claudius Balzer, Susan S. Eagle, Franz J. Baudenbacher, Matthias L. Riess

**Affiliations:** 1 Department of Anesthesiology, University Medical Center Göttingen, Robert-Koch-Str. 40, 37099 Göttingen, Germany; 2 Department of Anesthesiology, Vanderbilt University Medical Center, 1211 Medical Center Drive, Nashville, TN 37232 USA; 3 Department of Biomedical Engineering, Vanderbilt University, 2301 Vanderbilt Place, Nashville, TN 37235 USA; 4 Department of Anesthesiology, TVHS VA Medical Center, 1310 24th Ave S, Nashville, TN 37212 USA; 5 Department of Pharmacology, Vanderbilt University, 465 21St Avenue South, Nashville, TN 37240 USA

**Keywords:** End-tidal CO_2_, Fourier transformation, Monitoring, Murine, PEA, PIVA, Quality, Waveform analysis

## Abstract

**Background:**

Enhancing venous return during cardiopulmonary resuscitation (CPR) can lead to better hemodynamics and improved outcome after cardiac arrest (CA). Peripheral Intravenous Analysis (PIVA) provides feedback on venous flow changes and may indicate an increase in venous return and cardiac output during CPR. We hypothesize PIVA can serve as an early indicator of increased venous return, preceding end-tidal CO_2_ (etCO_2_) increase, before the return of spontaneous circulation (ROSC) in a rat model of CA and CPR.

**Results:**

Eight male Wistar rats were intubated and ventilated, and etCO_2_ was measured. Vessels were cannulated in the tail vein, femoral vein, femoral artery, and central venous and connected to pressure transducers. Ventilation was discontinued to achieve asphyxial CA. After 8 min, CPR began with ventilation, epinephrine, and automated chest compressions 200 times per minute until mean arterial pressure increased to 120 mmHg. Waveforms were recorded and analyzed. PIVA was calculated using a Fourier transformation of venous waveforms. Data are mean ± SE. Maximum PIVA values occurred in the tail vein 34.7 ± 2.9 s before ROSC, with subsequent PIVA peaks in femoral vein and centrally at 30.9 ± 5.4 and 25.1 ± 5.0 s, respectively. All PIVA peaks preceded etCO_2_ increase (21.5 ± 3.2 s before ROSC).

**Conclusion:**

PIVA consistently detected venous pressure changes prior to changes in etCO_2_. This suggests that PIVA has the potential to serve as an important indicator of venous return and cardiac output during CPR, and also as a predictor of ROSC.

## Background

In both the United States and Europe, emergency medical services respond to over 350,000 cases of adult out-of-hospital cardiac arrests (OHCA) every year [1, 2]. Despite best efforts, the survival rate of OHCA remains low at under 10%, with little improvement in the last decade [1].

Recent studies have tried to improve outcome after cardiac arrest (CA) by targeting venous return to enhance hemodynamics during cardiopulmonary resuscitation (CPR) with highly promising devices, such as active compression–decompression (ACD) CPR and the impedance threshold device [3–7]. However, it still remains difficult to quantify venous return during CPR, since the direction of venous blood flow changes between compression and decompression. Several studies related to the ongoing debate on the potential physiological mechanisms of CPR, such as the cardiac, thoracic, or pulmonary pump theories, agree that there is also retrograde venous flow during CPR. While older studies assume that retrograde flow is inhibited by collapse of the veins at the thoracic inlet and closure of the venous valves [8, 9], more recent studies show that retrograde venous pulsation may also continue in the peripheral vessels during compressions [10, 11]. In the present study, we examined the venous pulsation with Peripheral Intravenous Analysis (PIVA), a novel method for determining cardiac and volume status using waveforms from a standard peripheral intravenous (IV) line [12–16]. Based on our recent study, which demonstrated that high *central* venous pressure amplitudes (CVP-A) during CPR indicate improved outcome [17], we hypothesized that changes in *peripheral* venous amplitude, as measured by PIVA, could serve as an early indicator of increased venous return in a rat model of CA and CPR. Since there is currently no established marker for venous return, we explored this hypothesis in relation to the temporal progression of CVP-A and end-tidal CO_2_ (etCO_2_) levels, as one of the few recognized markers of high-quality CPR [18–21], and occurrence of return of spontaneous circulation (ROSC).

## Methods

This study is a post hoc analysis of the control group (8 animals) of a previously conducted study (unpublished). We were able to use the data for our exploratory and confirmatory study and, thus, comply with the 3Rs principle of humane animal research. The original study was approved by the Institutional Animal Care and Use Committee at Vanderbilt University Medical Center, Nashville, TN and was carried out and reported in accordance with the ARRIVE guidelines for animal research (Approval M1800029-00) [22].

Eight male Wistar rats 38.3 ± 1.5 weeks old and weighing 418.1 ± 45.6 g were housed under a 12/12 h light/dark cycle with water and food ad libidum. Immediately prior to the experiment, rats were anesthetized with an intraperitoneal (IP) pentobarbital injection (45 mg kg^−1^, Diamondback Drugs, Scottsdale, AZ, USA). Rats were intubated with a 14G angiocatheter videoendoscopically as previously described [23] and then mechanically ventilated with 6 mL kg^−1^ tidal volume, 60 breaths minute^−1^, 50% oxygen in nitrogen mixture (rodent ventilator model 683, Harvard Apparatus, MA, USA). IV pentobarbital was titrated to effect for maintenance of anesthesia. EtCO_2_ was measured by an infrared CO_2_-Sensor (Capnogard, Novametrix, CT, USA). A rectal temperature probe was placed, and core temperature maintained constant between 36.5 °C and 37.5 °C. Electrocardiogram (ECG) was derived from subcutaneous ECG needles to calculate heart rate (HR). Vascular access was established via surgical cut-down using a 22G 1″ angiocatheter (Smiths Medical, Minneapolis, MN, USA) in the right femoral vein, a 24G 1″ angiocatheter (Smiths Medical, Minneapolis, MN, USA) in the tail vein as well as blunt needles (Air-Tite, Virginia Beach, VA, USA) connected to 25 cm PE tubing (PE25 Instech, Plymouth Meeting, PA, USA) in the femoral artery and femoral vein, respectively. The left femoral venous catheter was advanced into the right atrium. A TruWave pressure transducer (Edwards Lifesciences, Irvine, CA, USA) was connected directly to each catheter. ECG, arterial pressure [mean arterial pressure (MAP), systolic and diastolic pressure], and all venous pressures (central venous pressure [CVP] and amplitude [CVP-A]) and waveforms were recorded using Powerlab Series 16/30 and LabChart Version 8.1.13 (AD Instruments, Dunedin, New Zealand). Coronary perfusion pressure (CPP) was calculated as the difference of diastolic blood pressure and minimal CVP during CPR [24]. Respiratory arrest was achieved by IV injection of rocuronium (3 mg kg^−1^, Hospira, Lake Forest, IL, USA) and discontinuation of ventilation for 8 min. Respiratory arrest was defined as an absence of respiratory effort and an absence of etCO_2_. This asphyxial arrest is typically described as an animal model of biventricular failure [25] and CA occurred within 3–4 min after cessation of ventilation. CA was defined as the absence of pulsatility on the arterial pressure tracing despite continued electrical activity on ECG (Pulseless Electrical Activity [PEA] arrest). At the conclusion of the apneic period of 8 min, ventilation was resumed (6 mL kg^−1^ tidal volume, 60 breaths per minute, 100% inspired oxygen concentration). Intravenous epinephrine was administered (0.01 mg kg^−1^, Hospira). Chest compressions were performed to a depth of 2 cm and a compression rate of 200 min^−1^ utilizing a custom-built piston compression device until a systolic blood pressure of 120 mmHg was achieved. At that point, compressions were stopped to evaluate for ROSC. ROSC was defined by return of spontaneous pulsatility on the arterial pressure tracing and maintenance of etCO_2_ > 20 mmHg in the absence of chest compressions. Ventilation continued and vital signs were allowed to stabilize after achieving ROSC.

Venous pressure waveform was analyzed offline after the conclusion of the experiment using a Fast Fourier Transform (FFT) with an 8 K sampling window with no window overlap. Data were recorded at a sampling rate of 1 kHz necessitating 8 s of continuous time-domain signal to perform the 8 K-FFT spectral analysis. The FFT converts the time-domain waveform into a frequency-domain output with distinct peaks at representing the respiratory and cardiac activity. In this study the dominant frequency corresponds to the speed of chest compressions (200 min^−1^ or 3.3 Hz) during CPR which we used to calculate the amplitude of the PIVA signal.

A significant increase in etCO_2_ was defined as an increase of more than 100% with respect to the first lowest value during CPR. This relative threshold was chosen due to the substantial variability in etCO_2_ values during CPR after asphyxial CA [26], while there is still no definitive value for a significant rise in etCO_2_ as a marker of ROSC [27]. All waveforms were recorded and analyzed in LabChart and MATLAB (Mathworks, Novi, MI, USA). To account for variations in CPR duration, the pressure amplitude values were segmented into quarters and averaged for presentation. To ensure that all data follow a normal distribution, a Shapiro–Wilk normality test was run. Data are expressed as mean ± standard error (SE) and uncorrected Fisher's LSD test with α = 0.05 was performed to test for significant differences in the time between the peak in PIVA and the 100% increase in etCO_2_ in relation to the time of ROSC as defined above. All calculations and graphics were performed using Graphpad Prism (Prism 9, GraphPad Software, LA Jolla, CA, USA), Python (Python Software Foundation. Python Language Reference, Version 3.8. Available at https://www.python.org. 2019) and Stata (Stata/IC 16.1, StataCorp LLC, College Station, TX, USA).

## Results

Eight animals were included in the experiment and successfully underwent the respiratory arrest procedure leading to PEA CA. Eight minutes after its cessation, ventilation was resumed, and all eight rats received IV epinephrine and chest compressions which resulted in successful ROSC within 54.6 ± 1.5 s.

During the entire CPR period, CVP-A demonstrated a steady increase. In contrast, the venous pressure amplitudes in the femoral and tail veins showed an initial increase followed by a subsequent decrease (Table 1). Furthermore, CVP exhibited higher amplitudes than those recorded in the femoral and tail veins.

**Table 1:** Hemodynamic overview

	First quarter	Second quarter	Third quarter	Fourth quarter
MAP (mmHg)	14.4 ± 0.7	22.7 ± 1.6	44.7 ± 2.0	81.7 ± 5.7
Systole (mmHg)	19.2 ± 1.2	30.4 ± 2.6	60.4 ± 4.0	110.5 ± 8.9
Diastole (mmHg)	13.1 ± 0.8	19.7 ± 1.1	38.6 ± 2.1	69.8 ± 4.5
etCO_2_ (mmHg)	28.9 ± 5.7	27.3 ± 3.8	37.0 ± 3.9	48.7 ± 3.6
CPP (mmHg)	13.1 ± 0.8	19.7 ± 1.1	38.6 ± 2.1	69.8 ± 4.5
Tail pressure (mmHg)	13.4 ± 0.6	12.7 ± 0.7	13.1 ± 0.3	12.7 ± 0.2
Femoral pressure (mmHg)	14.8 ± 0.5	13.9 ± 0.5	12.9 ± 0.5	12.5 ± 0.5
Central venous pressure (mmHg)	13.9 ± 0.8	13.5 ± 1.8	13.0 ± 2.2	12.7 ± 1.9
Tail venous amplitude (mmHg)	1.3 ± 0.8	1.7 ± 1.1	1.7 ± 1.2	1.4 ± 1.0
Femoral venous amplitude (mmHg)	4.1 ± 1.2	6.4 ± 1.7	6.1 ± 1.6	5.6 ± 1.5
Central venous amplitude (mmHg)	5.7 ± 1.0	9.5 ± 2.4	10.4 ± 2.7	10.8 ± 2.7

Arterial pressure and venous pressure amplitudes of tail vein, femoral vein, and central venous pressure over the CPR period divided into quarters of the entire CPR period. Data are mean ± SE mmHg. All statistical tests (Kruskal–Wallis and Mann–Whitney U tests) showed significant differences between quarters (p < 0.01)

**Fig. 1:**
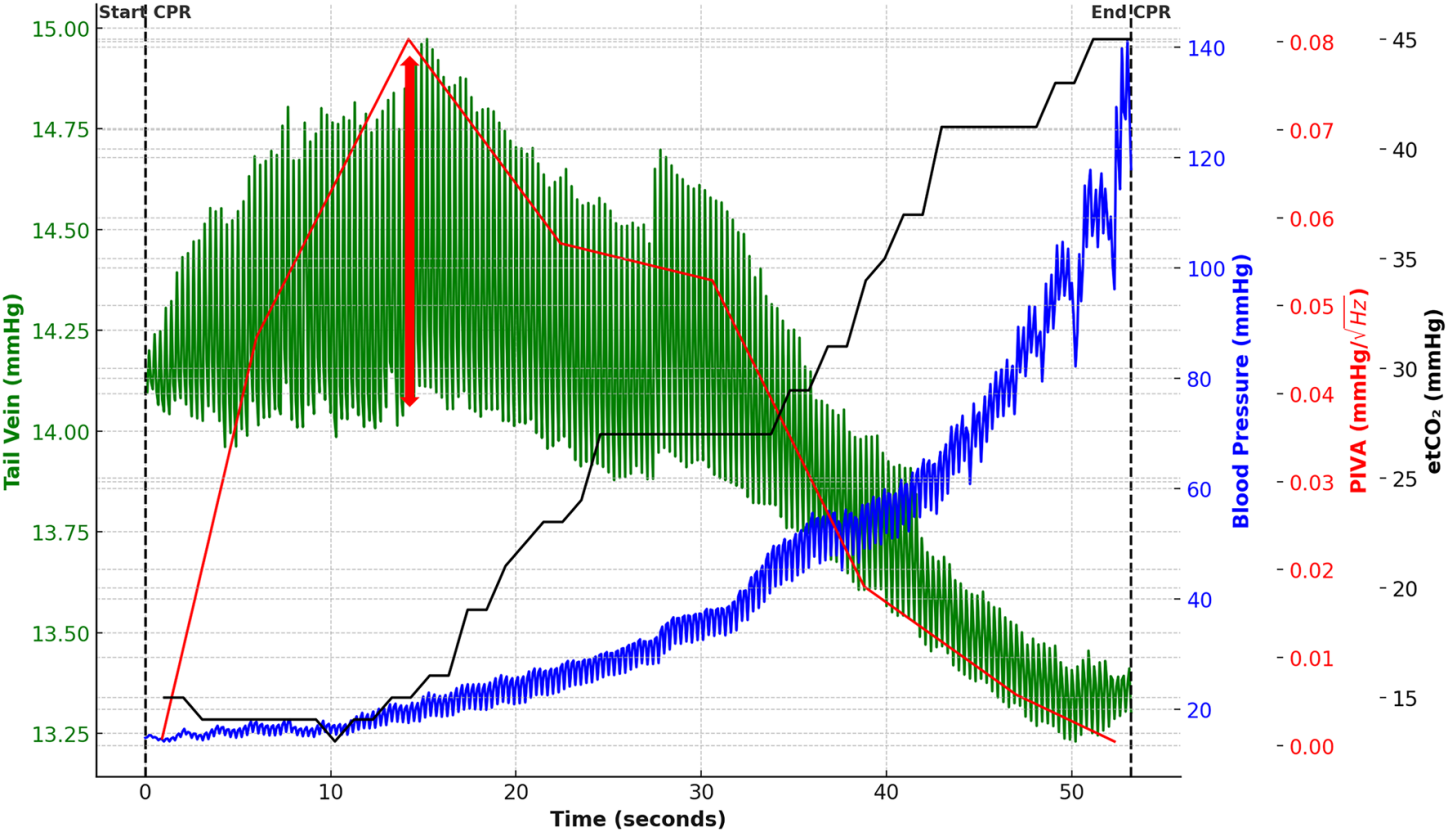
Exemplary visualization of PIVA, venous pressure, and vital parameters during CPR. Representative image of a peripheral venous waveform amplitude in the tail vein (green), along with the corresponding PIVA values (red), in context with blood pressure (blue) and etCO_2_ (black) over the time course of CPR. The maximum venous amplitude is indicated by the red arrow

**Fig. 2:**
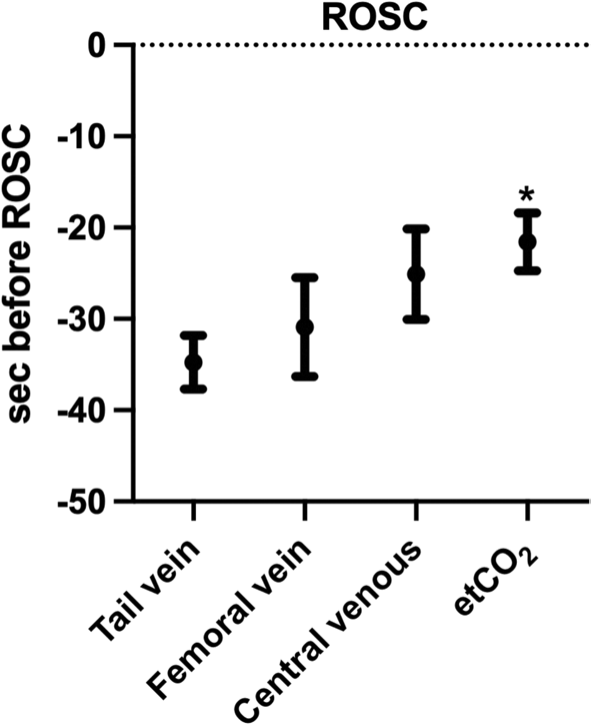
Timing of PIVA and ROSC. Timing of PIVA peaks of tail vein, femoral vein, and central venous pressure to etCO_2_ in relation to ROSC/end of chest compressions. Data are mean ± SE, * Tail vein vs. etCO_2_ p = 0.03

We observed an increase in PIVA at all sites with a single, prominent peak and subsequent decline (Fig. 1). The first peak of PIVA was seen in the tail vein 34.7 ± 2.9 s before ROSC, with subsequent PIVA peaks in femoral vein and central venous at 30.9 ± 5.4 and 25.1 ± 5.0, respectively (Fig. 2). In all experiments, at least one or all PIVA peaks were seen before an increase in etCO_2,_ that appeared 21.5 ± 3.16 s before ROSC, and exhibiting a statistically significant time difference compared to the tail vein (Fig. 2).

## Discussion

This study investigated the utility of PIVA during CPR in a rat model of asphyxial CA. Our results show that PIVA was able to detect an increase in venous return with subsequent increase in etCO_2_ followed by ROSC. Across all experiments, PIVA consistently exhibited sequential peaks in the tail vein, followed by the femoral vein and central venous site. Notably, all PIVA peaks occurred prior to the rise in etCO_2_ levels. These results suggest that a decrease in PIVA signal indicates achievement of a critical threshold of blood flow during CPR and might predict the occurrence of ROSC.

To the best of our knowledge, we are the first group to measure peripheral venous pressure and analyze its components during CPR. Previous studies described a retrograde venous flow during compressions, but only with the consequence that this palpable pulsation could be misleading for pulse checks or arterial cannulations [10, 11, 28]. This backflow phenomenon is reflected in our study with an early increase of venous amplitudes in all peripheral catheters, seen by increasing pressure amplitudes and calculated by PIVA values. However, as pressure amplitudes are decreasing during the course of CPR, PIVA detects a decrease of retrograde venous flow, when venous return increases and hemodynamics improve. These findings are also based on the fluid dynamic principles: If a pulsation travels toward a closed end of a tube, the amplitude increases due to reflection. Conversely, if the pulsation decreases and travels toward the open end, the amplitude decreases as there is no reflection.

Particularly important at this point is the CVP pulsation, previously defined as CVP-A, resulting in PIVA peaks short before ROSC: In a recent study, we were able to show that a high CVP-A during CPR was associated with a higher successful defibrillation rate, which underlines the notion that PIVA peaks could reflect venous return and a critical threshold of flow that improves the chances for ROSC and improved outcomes [17]. An important common feature of this investigation and the present study is the significance of the amplitude as opposed to the average value of the venous pressure. Just as the CVP as a static measurement has little significance for the outcome compared to the CVP-A, the significance of the peripheral venous pressure is hidden in the close observation of the peripheral venous pressure curve by PIVA and, thus, offers a promising dynamic parameter.

Furthermore, it is of great importance that this study, like the CVP-A study, describes changes in venous amplitudes significantly earlier than etCO_2_. While increased venous return and increased CO_2_ delivery to the lungs occur simultaneously after ROSC, there are physiologic reason for the delay in detecting ROSC by etCO_2_. First, the CO_2_ washout from the tissue after poor perfusion takes time [29]. Additionally, CO_2_ is delayed by circulatory factors due to the distance the blood must travel before reaching the lungs [30]. The release of CO_2_ by the lungs is further impaired due to uneven alveolar ventilation and a perfusion mismatch, before the CO_2_ can be detected by a capnometer [29].

One crucial aspect is that etCO_2_ measurements can only be reliably obtained from patients who are intubated and ventilated using equipment capable of CO_2_ monitoring. Conversely, PIVA holds the potential for widespread clinical utilization as it relies on the simple procedure of peripheral intravenous cannulation, which is highly recommended for all patients undergoing CPR [1].

Another indicator for the significance of PIVA, as the earliest indicator of a critical threshold of blood flow before ROSC, are the values of other hemodynamic parameters at the time point of the PIVA peak (Table 2). At this timepoint exhibits none of the values, that could be predictive for ROSC, provide clinical forecasting values in context with the values over time (Table 1).

**Table 2:** Hemodynamics at the timepoint of the PIVA peak

MAP (mmHg)	19.5 ± 1.9
Systole (mmHg)	26.8 ± 1.9
Diastole (mmHg)	17.3 ± 1.8
CVP (mmHg)	12.9 ± 2.3
CVP-A (mmHg)	8.9 ± 2.4
CPP (mmHg)	8.1 ± 2.3
etCO_2_ (mmHg)	25.4 ± 3.1

*MAP* Mean arterial pressure, *CVP and CVP-A* Central venous pressure and amplitude, *CPP* coronary perfusion pressure at the time of the PIVA peak. Data are mean ± SE mmHg

With further development of this parameter into a device with immediate feedback, a novel instrument could offer significant benefits by providing immediate insights into venous return and the quality of CPR, which is critical for guiding medical personnel during resuscitation.

The results of this study, however, need to be evaluated within their natural constraints. While real-time analysis has the potential to improve CPR, our study was a retrospective analysis, which could only identify the PIVA peak and subsequent fall in retrospect. Furthermore, the sample size was small in this feasibility study, and more studies with different CA modalities will be needed to confirm our findings. In particular, we only examined one CA etiology. While asphyxial CA leads to heart failure with consecutive venous fluid overload, PIVA signals after sudden CA with, e.g., ventricular fibrillation could detect different venous waveforms during CPR. Furthermore, the etCO_2_ threshold needs to vary depending on the etiology of cardiac arrest to fully realize the potential of PIVA, as different arrest mechanisms, such as asphyxial versus fibrillatory, result in significantly different etCO_2_ dynamics. Another limitation is the absence of a control group without ROSC. This limitation is caused by the rodent asphyxial cardiac arrest model itself, since omitting or delaying catecholamines is not a safe method of avoiding ROSC, and there is evidence that ROSC can also occur without epinephrine in this model [31, 32]. As a next step, we will not only test PIVA in different CA models, but we are also preparing a clinical study to test PIVA during CPR in the field.

## Conclusions

In this study, PIVA was able to detect venous return and predict subsequent ROSC in a rat model of asphyxial CA and CPR. Thus, PIVA could potentially serve as a novel indicator of resuscitation efficacy and success.

## Data Availability

The datasets used and analyzed during the current study are available from the corresponding author on reasonable request.

## Abbreviations

ACD: Active compression–decompression
CA: Cardiac arrest
CPR: Cardiopulmonary resuscitation
CPP: Coronary perfusion pressure
CVP: Central venous pressure
CVP-A: Central venous pressure amplitudes
etCO_2_: End-tidal CO_2_
FFT: Fast Fourier transform
IV: Intravenous
OHCA: Out-of-hospital cardiac arrests
PIVA: Peripheral intravenous analysis
ROSC: Return of spontaneous circulation
